# Plasticity of Naturally Occurring Regulatory T Cells in Allergic Airway Disease Is Modulated by the Transcriptional Activity of *Il-6*

**DOI:** 10.3390/ijms22094582

**Published:** 2021-04-27

**Authors:** Morgan MacBeth, Anthony Joetham, Erwin W. Gelfand, Michaela Schedel

**Affiliations:** 1 Division of Allergy and Immunology and Cell Biology, Department of Pediatrics, National Jewish Health, Denver, CO 80206, USA; morgan.macbeth@cuanschutz.edu (M.M.); joethama@njhealth.org (A.J.); erwin3460@gmail.com (E.W.G.); 2 Department of Medical Oncology, University of Colorado, Denver, CO 80206, USA; 3 Department of Pulmonary Medicine, University Medical Center Essen-Ruhrlandklinik, 45239 Essen, Germany; 4 University Hospital Essen, University Duisburg-Essen, 45147 Essen, Germany

**Keywords:** IL-6, naturally occurring T regulatory cells (nTregs), plasticity, transcriptional regulation, transcription factor binding

## Abstract

The impact of naturally occurring regulatory T cells (nTregs) on the suppression or induction of lung allergic responses in mice depends on the nuclear environment and the production of the pro-inflammatory cytokine interleukin 6 (IL-6). These activities were shown to be different in nTregs derived from wild-type (WT) and CD8-deficient mice (CD8^−/−^), with increased IL-6 levels in nTregs from CD8^−/−^ mice in comparison to WT nTregs. Thus, identification of the molecular mechanisms regulating IL-6 production is critical to understanding the phenotypic plasticity of nTregs. Electrophoretic mobility shift assays (EMSA) were performed to determine transcription factor binding to four *Il-6* promoter loci using nuclear extracts from nTregs of WT and CD8^−/−^ mice. Increased transcription factor binding for each of the *Il-6* loci was identified in CD8^−/−^ compared to WT nTregs. The impact of transcription factor binding and a novel short tandem repeat (STR) on *Il-6* promoter activity was analyzed by luciferase reporter assays. The *Il-6* promoter regions closer to the transcription start site (TSS) were more relevant to the regulation of *Il-6* depending on NF-κB, c-Fos, and SP and USF family members. Two *Il-6* promoter loci were most critical for the inducibility by lipopolysaccharide (LPS) and tumor necrosis factor α (TNFα). A novel STR of variable length in the *Il-6* promoter was identified with diverging prevalence in nTregs from WT or CD8^−/−^ mice. The predominant GT repeat in CD8^−/−^ nTregs revealed the highest luciferase activity. These novel regulatory mechanisms controlling the transcriptional regulation of the *Il-6* promoter are proposed to contribute to nTregs plasticity and may be central to disease pathogenesis.

## 1. Introduction

IL-6 acts as a pro-inflammatory cytokine with a critical role in immune responses [1]. Impaired regulation of IL-6 can lead to immune-related diseases including rheumatoid arthritis, where increased IL-6 levels were observed, and has thus been used as a therapeutic target [2]. While *IL-6* knockout mice have also been shown to present with heightened inflammatory responses with regional tissue damage [1,3], an excess of IL-6 is linked to Castleman’s disease [4], indicating the importance of the appropriate regulation of IL-6 levels. IL-6 has also emerged as a pivotal factor in the pathogenesis of asthma [5,6,7], a heterogeneous syndrome involving complex pathophysiological pathways. In addition to IL-6, soluble IL-6 receptor (sIL-6R) levels have consistently been elevated in children and adults with asthma [5,6,7,8,9,10]. Levels were positively correlated with disease severity and inversely correlated with forced expiratory volume in 1 s (FEV1). T regulatory cells (Tregs), including naturally occurring T regulatory cells (nTregs, CD4^+^CD28^high^Foxp3^+^) derived from the thymus are central to maintaining immune homeostasis, a balance that is altered in asthma [11]. Importantly, the functional activities of this regulatory subset are not fixed but display significant plasticity under different environmental conditions. Whereas WT nTregs have been shown to effectively prevent airway allergic inflammation and improve airway function in allergen-sensitized and challenged WT recipients [12], these same nTregs enhanced lung allergic responses when transferred into CD8^−/−^ mice [13]. Conversely, nTregs from CD8^−/−^ mice exhibited lower suppressor activity after adoptive transfer into sensitized and challenged WT recipient mice [14]. Central to these differing activities appeared to be levels of IL-6, as nTregs from CD8^−/−^ mice produced significantly higher IL-6 levels in comparison to nTregs from WT mice. Indeed, the suppression of lung allergic responses was restored after administration of anti-IL-6 in WT recipients prior to the adoptive transfer of CD8^−/−^ nTregs [14]. The importance of IL-6 production on nTreg function was further strengthened as nTregs from IL-6-deficient mice were suppressive when transferred into sensitized and challenged WT recipients. However, suppressor activity was lost when pretreated with IL-6 prior to transfer. These findings underscore the need to better understand the molecular regulation of *Il-6* in dictating nTreg plasticity, leading to the conversion from suppressive to pathogenic effector cells. To this end, we characterized and compared crucial regulatory regions within the *Il-6* promoter gene in nTregs from WT and CD8^−/−^ mice, regions which governed the differential expression of IL-6 and contributed to their suppressive or enhancing activities depending on their nuclear environment. Differences in the binding intensity of transcription factors including NF-κB, c-Fos, and family members of the specificity protein (SP) and upstream stimulatory factor (USF) were identified in the *Il-6* promoter in both two strains. These differences influenced the in vitro promoter activity of *Il-6* in response to LPS and TNFα stimulation. In addition, a novel STR within the *Il-6* promoter region was characterized, relevant to the distinct regulation of *Il-6* in nTregs from WT and CD8^−/−^ mice.

## 2. Results

### 2.1. Transcription Factor Binding to the Il-6 Promoter Is Increased in nTregs from CD8^−/−^ Mice

#### 2.1.1. Human and mouse *IL-6* promoter share high homology

Using the Visualization Tool for Alignment (VISTA) Genome Browser database [15,16], the human *IL-6* and mouse *Il-6* promoter revealed up to 95% homology (Online Supplemental Figure S1A). The region of the *Il-6* promoter directly upstream of the TSS was more conserved between human and mice in comparison to its corresponding exons. nTregs from CD8^−/−^ mice produce significantly higher amounts of IL-6 than WT nTregs [14]. To identify *cis-*regulatory elements potentially contributing to the differential expression of IL-6 levels in nTregs from WT and CD8^−/−^ mice and their distinct suppressive capacities [13,14], *in silico* transcription factor binding analyses of approximately 500 bp upstream of the TSS of the mouse *Il-6* promoter were performed. The *Il-6* promoter region in mice revealed a sequence homology to human *IL-6* of 71% (Online Supplemental Figure S1B). Using the MatInspector software tool [17], a total of 126 potential transcription factor binding sites were identified (Online Supplemental Table S1). Consistent with previous reports [18,19,20,21,22], this included predicted binding sites for NF-κB, AP-1, and C/EBP, which were highly homologous in humans (Online Supplemental Figure S1A,B).

#### 2.1.2. Elevated Binding of NF-κB, SP1, and SP3 in nTregs from CD8^−/−^ Compared to WT Mice

To delineate formed DNA/protein interactions specific to nTregs, these three loci in the mouse *Il-6* promoter upstream of the TSS homologous to human *IL-6* were analyzed. They were suggested to influence its regulatory function (Online Supplemental Figures S1B and S2) when examined by EMSA with nuclear extracts isolated from nTregs of WT and CD8^−/−^ mice. A previously uncharacterized region within the mouse *Il-6* promoter, most distinct from human *IL-6* (Online Supplemental Figure S1B), was investigated (Il-6_4). Each potential binding site under study was numbered in accordance with the relative distance to the TSS, respectively (Il-6_1, Il-6_2, Il-6_3, and Il-6_4, see Online Supplemental Figure S2).

**Figure 1:**
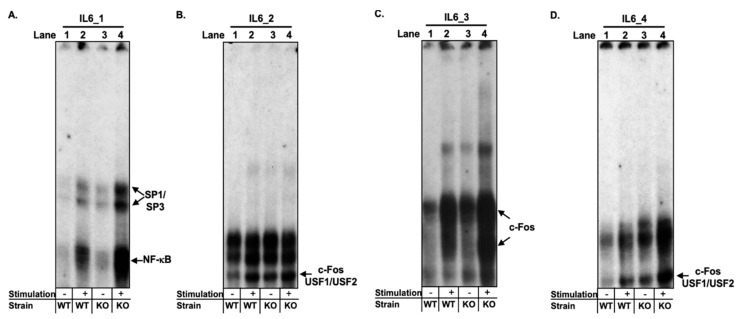
Transcription factor binding to promoter regions within the *Il-6* gene is increased in nTregs from CD8^−/−^ mice compared to wild-type (WT) nTregs. (**A**–**D**). EMSA analyses of four different *Il-6* promoter loci upstream of the transcription start site of the *Il-6* gene were performed, including (**A**) Il-6_1, (**B**) Il-6_2, (**C**) Il-6_3, and (**D**) Il-6_4 using nuclear extract isolated from nTregs from WT or CD8^−/−^ (KO) mice (5 µg) cultured for 3 h either left in medium (−) or stimulated (+) with PMA/ionomycin (50 ng/mL/1 µM).

For the region closest to the TSS of *Il-6* (Il-6_1), a number of complexes were identified with an overall higher binding in unstimulated and stimulated (phorbol 12-myristate 13-acetate (PMA)/ionomycin) nuclear extracts from CD8^−/−^ nTregs compared to WT nTregs (Figure 1A, lanes 3 and 4 vs. 1 vs. 2). To verify the predicted binding of NF-κB, competition experiments with a high affinity NF-κB consensus site (100-fold molar excess) [23,24] were performed, leading to the abrogation of all complexes (Online Supplemental Figure S3A, lane 3). A DNA/protein complex was confirmed to selectively contain the NF-κB subunit p65 (Online Supplemental Figure S3A, lane 7), while no binding of the p50 subunit was observed (lane 6). A novel protein/DNA interaction of transcription factors of the specificity protein (SP) family including SP1 and SP3 in the nuclear environment of nTregs was identified. Their binding to Il-6_1 was confirmed by competition experiments with a SP consensus site [25,26] and using specific antibodies (Online Supplemental Figure S3A, lanes 4 and 8–11). Overall, the locus closest to the TSS of *Il-6* showed greater binding of p65 and SP family members in CD8^−/−^ nTregs compared to WT nTregs before and after stimulation.

#### 2.1.3. Three Loci in the *Il-6* Promoter Revealed Increased Transcription Factor Binding Signaling for c-Fos, USF1, and USF2 in nTregs from CD8^−/−^ Mice

Stronger transcription factor binding patterns to different sequence regions within the *Il-6* promoter were present for three additional potential regulatory elements in unstimulated and stimulated CD8^−/−^ nTregs compared to WT nTregs (Figure 1B–D). In contrast to the *in silico* prediction of C/EBP binding to Il-6_2 (Online Supplemental Figure S2, Online Supplemental Table S1), a supershift for c-Fos, USF1, and USF2 using specific antibodies (Online Supplemental Figure S3B, lanes 6, 8, and 9) was seen with a stronger complex formation signal using nuclear extracts from unstimulated nTregs from CD8^−/−^ mice (Figure 1B, lanes 1 vs. 3). The specific binding of these transcription factors was confirmed by competition experiments with an AP1 [26] and USF [27] consensus site (Online Supplemental Figure S3B, lanes 3 and 4).

Increased protein/DNA binding in nTregs from CD8^−/−^ mice in contrast to WT nTregs before and after stimulation was also seen for Il-6_3 (Figure 1C, lanes 3 and 4 vs. 1 and 2). As suggested by the prediction model (Online Supplemental Figure S2, Online Supplemental Table S1), binding of AP1 to Il-6_3 was verified but only for the c-Fos subunit (Online Supplemental Figure S3C, lanes 3 and 6), independently of c-Jun (Online Supplemental Figure S3C, lane 7). No specific complex formation of USF1 and USF2 to this *Il-6* promoter region was detected (Online Supplemental Figure S3C, lanes 8 and 9).

Similarly, the transcription factors involved as potential regulatory elements most distant to the TSS of *Il-6* (Il-6_4) included c-Fos, USF1, and USF2 (Online Supplemental Figure S3D, lanes 6, 8, and 9). The DNA/protein complex formation intensity significantly increased using nuclear extracts from CD8^−/−^ compared to WT nTregs (Figure 1D, lanes 3 and 4 vs. 1 and 2). Taken together, using nuclear extracts from nTregs of CD8^−/−^ mice revealed an elevated transcription factor binding signal for all four *Il-6* DNA sequence regions under study (Figure 1A–D). This may contribute to the previously observed higher IL-6 levels in nTregs from these mice [14].

### 2.2. Defined Regions Are Vital for LPS- and TNFα-Mediated Il-6 Promoter Activation

**Figure 2:**
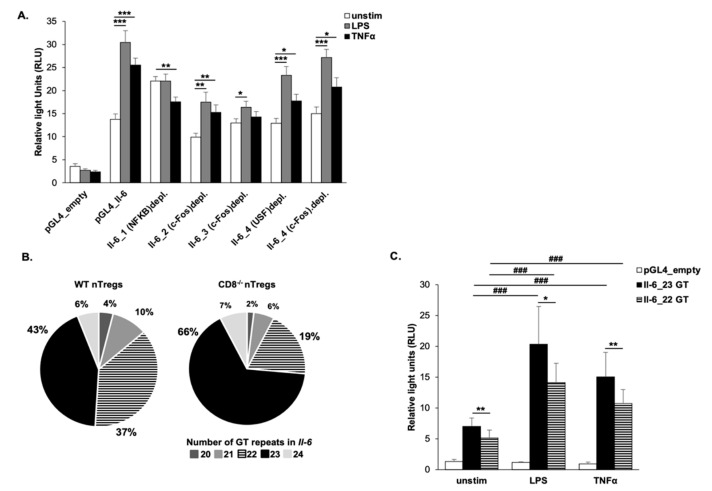
Transcription factor binding sites of NF-κB, c-Fos, and members of the SP and USF family as well as a novel GT repeat affect *Il-6* promoter activity. (**A**) NIH3T3 cells were transiently transfected with the empty pGL4 luciferase vector (pGL4 empty) or with different reporter constructs of the *Il-6* promoter (1138 bp). In the construct Il-6_1 (NF-κB)depl., the NF-κB binding site as identified by EMSA within Il-6_1 was depleted by site-directed mutagenesis. Correspondingly, the c-Fos binding site within Il-6_2 (Il-6_2(c-Fos)depl.), the c-Fos binding site within Il-6_3 (Il-6_3(c-Fos)depl.), the USF binding site within Il-6_4 (Il-6_4(USF)depl.), and the c-Fos binding site within Il-6_4 (Il-6_4(c-Fos)depl.) were mutated (4–5 independent experiments, 2 technical replicates). Cells were left in medium or stimulated with 10 ng/mL LPS or 0.5 ng/mL TNFα. (**B**) *Il-6* luciferase promoter constructs were generated using DNA isolated from WT or CD8^−/−^ nTregs. To determine the frequency of a GT short tandem repeat in nTregs of both strains, a total of 104 clones (WT = 51, CD8^−/−^ = 53) were sequenced. The percentage of each of the detected GT STRs in WT or CD8^−/−^ nTregs is depicted (**C**) NIH3T3 cells were transiently transfected with the empty pGL4 luciferase vector or with reporter constructs of the *Il-*6 promoter region containing 22 or 23 GT repeats. Cells were left in the medium or stimulated with 10 ng/mL LPS or 1 ng/mL TNFα. For all luciferase experiments, luciferase activity was normalized for transfection efficiency using the control plasmid pRL-TK. The relative luciferase activity is presented in relative light units (RLU). Paired, two tailed students t-test was performed. * *p* ≤ 0.05, ** *p* ≤ 0.01, *** *p* ≤ 0.001; *p*-value comparing different stimulation conditions: ### *p* ≤ 0.001.

To determine whether these transcription factors acted as regulatory elements of *Il-6*, plasmids containing the firefly luciferase gene under the transcriptional control of the *Il-6* promoter were generated (Online Supplemental Figure S1B). We first confirmed that the *Il-6* promoter activity was highly inducible in response to LPS or TNFα stimulation in a mouse fibroblast cell line (NIH3T3 cells, Figure 2A). Each of the binding sites analyzed by EMSA was then individually depleted by inserting a point-mutation. At baseline, the *Il-6* promoter activity significantly increased after disruption of the binding sites for p65 and members of the SP family (Il-6_1, Online Supplemental Figure S4A). This introduced the full loss of the induction of the *Il-6* promoter in response to LPS and TNFα stimulation (*p* < 0.01, Figure 2A, Online Supplemental Figure S4A).

The mutation of the transcription factor binding sites for c-Fos and USF1/2 within Il-6_2 led to significantly lower baseline promoter activity compared to the *Il-6* wild-type luciferase construct (*p* < 0.01, Online Supplemental Figure S4A). When treated with LPS or TNFα, the *Il-6* promoter activity was still inducible, albeit to a lower degree (Figure 2A).

The depletion of the c-Fos/USF binding site in Il-6_3 did not influence baseline *Il-6* activity (Online Supplemental Figure S4A), but resulted in a loss of induction after stimulation with LPS or TNFα (Figure 2A).

In contrast, the independent depletions of the transcription factor binding sites including c-Fos and USF1/2 most upstream of the *Il-6* TSS (Il-6_4) had no discernable effect on the overall luciferase signal (Online Supplemental Figure S4A, Figure 2A), suggesting that this region may not be relevant for the transcriptional regulation of the *Il-6* promoter at baseline. However, the region furthest from to the TSS had the capacity to react to extrinsic stimuli including LPS and TNFα, mainly mediated through USF1/2 as the depletion led to a significantly lower inducibility of the *Il-6* promoter (Online Supplemental Figure S4A). Overall, each of the loci within the *Il-6* promoter acted as a transcriptional regulator with distinct binding signals of specific transcription factors including NF-κB, c-Fos, and members of the SP and USF family, thus potentially promoting the previously observed elevated IL-6 levels in nTregs from CD8^−/−^ mice in comparison to WT nTregs [14].

### 2.3. A Novel Short Tandem Repeat (STR) Influences Mouse Il-6 Promoter Activity

Within 500 bp upstream of the *Il-6* TSS (Online Supplemental Figure S2), we identified a novel variable STR (microsatellite) in nTregs ranging from 20 to 24 GT repeats in length. Following sequencing of 104 *Il-6* luciferase clones originating from nTregs from WT (*n* = 51 clones) or CD8^−/−^ (*n* = 53 clones) mice, the STR with 23 GT repeats was most common in both strains, with 66% and 43%, respectively (Figure 2B). This repeat was found to occur with a 3.5-fold increase in CD8^−/−^ nTregs compared to the 22 GT repeat while in nTregs from WT mice, only a marginal difference between these two STRs was detectable (Online Supplemental Figure S4B). The luciferase construct containing the 23 GT STR resulted in a significantly (*p* < 0.01) increased signal under the control of the *Il-6* promoter in unstimulated cells in comparison to the plasmid with 22 GT repeats (Figure 2C). Furthermore, the *Il-6* promoter signal was significantly higher in response to LPS and TNFα stimulation in the presence of 23 compared to 22 GT repeats (Figure 2C). These data suggest that the GT STR was associated with the transcriptional regulation of the *Il-6* promoter, providing a novel transcriptional mechanism explaining the observed increases in *Il-6* expression and the consequent failure of nTregs from CD8^−/−^ mice to suppress lung allergic responses [14].

## 3. Discussion

In models of experimental asthma, the plasticity and loss of suppressive activities of nTregs from CD8^−/−^ mice compared to WT mice was largely dependent on levels of IL-6 production [14,28]. The underlying molecular mechanisms controlling *Il-6* in nTregs have not been characterized to date. Here, we show that *Il-6* in nTregs from WT and CD8^−/−^ mice is regulated through alterations of the binding of a number of transcription factors which was related to the response of *Il-6* induction to external stimuli. Moreover, a novel STR was identified in the *Il-6* promoter, significantly influencing its activity. These studies emphasized that differential expression of IL-6 is complex and is mediated by several *cis*-acting regulatory elements, thus contributing to the distinct role of nTreg function.

**Figure 3:**
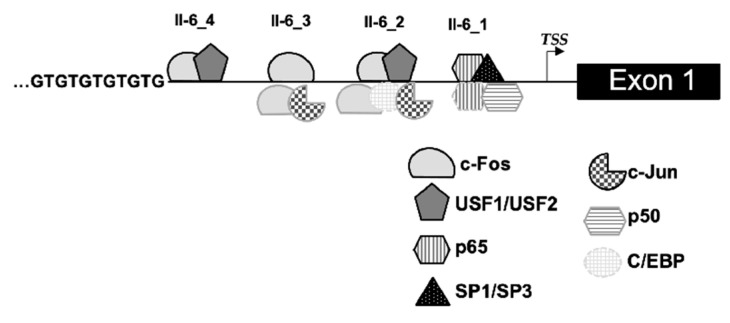
Schematic diagram of transcription factor binding to the *Il-6* promoter region in nTregs (upper panel) compared to previously published findings (lower panel).

IL-6 participates in a broad range of biological events and has been shown to engage in key processes to promote inflammatory diseases [1,3]. In asthmatics, IL-6 levels were significantly elevated in plasma [7], bronchoalveolar lavage fluid [8], and sputum [5,6,9] and correlated with disease severity [5,6,7]. IL-6 production was also elevated by viral infections and obesity [29,30,31], two important comorbid factors resulting in asthma exacerbations and severity. In patients with adult-onset asthma, elevated IL-6 was associated with high-dose inhaled corticosteroid use, systemic inflammation, and was linked to poor asthma control [32]. Outside of its role during disease, infection or stress, IL-6 is present at low expression levels [33]. Due to its many pleiotropic effects, IL-6 production is therefore tightly controlled. This complex regulation is cell type-specific and can vary in the same cell type depending on the stimuli [34]. As a result, it is essential that the transcriptional activities of *IL-6* are studied in the distinct nuclear environment. In order to better understand the transcriptional regulation of *Il-6,* we performed transcription factor binding analyses in nTregs from WT and CD8^−/−^ mice as well as promoter mapping. As summarized in Figure 3, a total of four sites were studied, some of which have previously been shown to contribute to *IL-6* regulation in different human and mouse cell lines [1,18,19,20,21,22]. To our knowledge, this is the first study investigating regulatory mechanisms of *Il-6* in primary nTregs comparing different mouse strains. Of note, across all four loci within the *Il-*6 promoter, increased occupation of transcription factor binding in nTregs from CD8^−/−^ compared to WT mice was seen at baseline and was enhanced in response to PMA and ionomycin, stimuli leading to the production of a variety of cytokines and a strong T cell activation [35]. The elevated binding signal using nuclear extracts from nTregs from CD8^−/−^ could be the result of overall increased binding affinity or a higher abundance of these transcription factors in the nucleus of CD8^−/−^.

Intact NF-κB binding in primary nTregs to a region closest to the TSS of *Il-6* was essential for its transcriptional regulation, as previously seen in different cell lines [19,20,21,36]. Concomitantly, we identified a novel DNA/protein interaction of members of the SP family including SP1 and SP3 to the same binding site. This region was required for significant induction of the *Il-6* promoter as one potential mechanism contributing to the observed increase in IL-6 levels in nTregs from CD8^−/−^ compared to WT mice [14]. Given the limitation of the unfavorable *ex vivo* expansion properties of nTregs, these promoter expression studies were performed in NIH3T3 cells, a cell line of choice known to express IL-6 with a functional IL-6 signal transduction pathway [37,38,39]. In nTregs, SP1 not only influences *Il-6* levels but also acts as a central element controlling the function of nTregs in mice due to its binding upstream of a *Foxp3* enhancer element [40]. Further upstream within the *Il-6* promoter, SP1 contributed to the transcriptional regulation in a mouse fibroblast B cell line, thus serving as a potential bridge to interact with NF-κB [36]. SP1 has been shown to bind with high affinity to specific NF-κB sites across the genome which were suggested to be mutually exclusive [41], while others observed a direct interaction between NF-κB and SP1 in a lung epithelial cell line [42]. Our data in nTregs support the latter. As NF-κB is fundamental in the regulation of *IL-6* in a number of different cell types, it is postulated that NF-κB is crucial for the constitutive expression of *Il-6*, while the interaction with other transcription factors including AP1 [41] or SP1/SP3 is part of a program to control precise patterns of *Il-6* in a distinct cell type. Although the binding of C/EBP was relevant for the transcriptional regulation of *IL-6* in cell lines [18,20,21,43,44,45], for example in concert with the NF-κB binding site [21], the ability to bind C/EBP, at least in nTregs from mice, was not required.

In addition, c-Fos may play a vital role to promote *Il-6* as c-Fos binding was observed to three *cis*-regulatory elements within the *Il-6* promoter, one of which has previously not been investigated. C-Fos is a subunit of the AP-1 complex forming homodimers as well as heterodimers with c-Jun [46]. The phosphorylation of c-Jun is mediated through the activation of the c-Jun N-terminal kinase (JNK) which in turn enables the interaction of c-Fos JunB, JunD, and ATF leading to the AP-1 complex formation [47]. Earlier studies in mouse cell lines demonstrated an involvement in the transcriptional regulation of *Il-6* for both AP-1 subunits, c-Fos and c-Jun [18,20]. In nTregs, the protein complex binding to the mouse *Il-6* promoter consisted only of the c-Fos subunit. This was surprising as JNK2 was identified to be essential in the enhancement of lung allergic responses mediated through nTregs, as demonstrated by the complete absence of exacerbating asthma-like immunopathology in sensitized and challenged WT mice following transfer of nTregs from JNK2-deficient mice [28]. It is therefore suggested that in nTregs, other regions within the *Il-6* promoter are controlled by c-Jun. The depletion of the c-Fos binding in Il-6_2 significantly decreased the promoter activity of *Il-6* in unstimulated cells, which was not observed for Il-6_3 and Il-6_4, suggesting that the transcriptional regulation of *Il-6* mediated through c-Fos binding closest to the transcription starts was more relevant for the baseline *Il-6* expression. Concomitant binding of c-Fos with members of the USF family (USF1/USF2) was detected for two regulatory elements within *Il-6*. USF is ubiquitously expressed and is involved in the transcription of a wide variety of genes, including the asthma susceptibility genes ORMDL sphingolipid biosynthesis regulator 3 *(ORMDL3)* [48] and plasminogen activator inhibitor 1 [49]. USF family members are part of helix-loop-helix proteins capable of interacting with enhancer box elements regulating target promoters [50,51]. For the binding site Il-6_4, the *in silico* model predicted an enhancer box binding region for which a protein/DNA complex formation of USF1/USF2 together with c-Fos was detected.

Based on our observations, the transcription factors NF-κB, c-Fos, SP1/SP3 and USF1/USF2 were found in a highly activated state, specifically in nTregs from CD8^−/−^ mice. Disrupting the binding sites within each of the four regulatory regions under study significantly decreased the *Il-*6 promoter activity in response to LPS and TNF**α**. LPS stimulates cells through Toll like receptor 4 signaling [52] mimicking an inflammatory response necessary for the activation of *Il-6* in nTregs [53]. TNF**α** is a pro-inflammatory cytokine that can downregulate the suppressive capacity of nTregs [54], which in turn leads to increased IL-6 levels. In contrast to USF1/USF2, mutating the cFos binding site within Il-6_4 showed little effect on the ability to activate the *Il-6* promoter, suggesting that the presence of this c-Fos regulatory site is not necessarily required. Overall, we believe that a complex interaction of these transcription factors is sufficient at basal level, but most importantly to the induction of IL-6, specifically in nTregs from CD8^−/−^ mice [14].

We discovered a novel STR at variable length in nTregs from WT and CD8^−/−^ mice upstream of the TSS of *Il-6.* Due to their association with human disease including asthma and atopic disease [55,56,57], the biological contribution of STRs has been studied intensively, leading to new paradigms to improve our current understanding of their effect on genome structure and function [58]. GT microsatellites are the second most common simple STR in the human genome within 5 kb of the TSS [55]. The prevalence of STRs including GT repeats specifically in regulatory regions, as seen in different organisms, suggests that repeat variation might be a common, evolutionarily conserved, mechanism regulating gene expression, thus supporting our findings. In contrast to the function of STRs in the predisposition to human disease, their role in disease-related mouse models including experimental asthma have to date been less well-characterized. Although the *IL-6* promoter region between human and mouse is homologous and has some overlapping regulatory elements in both species, this microsatellite was not present in the human *IL-6* promoter. The degree of conservation significantly declines directly upstream of this repeat suggesting the potential for evolutionary adaptation in humans as previously shown for other tandem repeats [59]. Noteworthy, within the same region of the human *IL-6* promoter, a base composition of a polyA (*n* = 8) followed by a polyT (*n* = 13) was present. It remains to be determined if this is a repetitive element of variable length with a mechanistic effect on the regulation of *IL-6* gene expression, thus contributing to human disease.

Amongst the detected number of repeats, 22 and 23 GT repeats were most frequent in nTregs from WT and CD8^−/−^ mice. Of note, the variation in length of this GT repeat significantly affected the promoter activity of *Il-6*. A similar effect has been described for the *STAT6* gene, critical to the Th2 cytokine signaling cascade [56]. A GT repeat influencing the regulation of the promoter activity of *STAT6* was associated with susceptibility to atopic asthma and total serum IgE levels. Given that the STR with 23 GT repeats led to the highest induction of the *Il-6* luciferase signal without or with stimulation and was most commonly found in nTregs from CD8^−/−^ in comparison to nTregs from WT mice, we believe that this microsatellite may be a novel mechanism contributing to the increased IL-6 production observed in CD8^−/−^ nTregs. One potential model to additionally explain our findings is through alterations of the chromatin architecture of *Il-6*, as seen for other disease-related STRs in close proximity to boundaries of CpG islands [58]. As this novel GT repeat is located only 10 bp upstream of a potential CpG island, this hypothesis opens up future studies aimed at elucidating the cause-and-effect relationship between STR, epigenetic changes, and the *cis*-regulation on the *Il-6* promoter in nTregs from CD8^−/−^ compared to WT mice.

There is compelling evidence of a phenotypic and functional instability of nTregs [60,61,62] which, amongst other factors [28,63,64,65], is related to shifted production of IL-6 specifically in nTregs from WT and CD8^−/−^ mice [14,28]. Suppressive nTregs were shown to be capable of converting in vivo into pathogenic cells, enhancing the full spectrum of lung allergic responses. To this end, we described underlying molecular mechanisms in *Il-6* controlling this conversion. Transcription factors close to the TSS directly interacted with the *Il-6* promoter in nTregs, influencing its activity, and responded to inflammatory stimuli. The binding signal of these transcription factors was distinct in nTregs from WT and CD8^−/−^ mice. The expansion of a newly identified STR in nTregs from CD8^−/−^ mice increased the transcriptional activity of *Il-6*. Taken together, these findings exhibit alternative regulation of *Il-6* in nTregs with distinct functions contributing to their plasticity and ability to undergo conversion, thereby influencing lung allergic responses and may be other IL-6-related diseases impacted by the nTregs.

## 4. Materials and Methods

### 4.1. In Silico Transcription Factor Binding Analyses

The MATinspector software tool (Genomatrix Suite, Precigen Bioinformation, Munich, Germany, www.genomatrix.de, accessed on 23 March 2021) was used to predict putative binding sites for transcription factors within 500 bp upstream the translation start site of *Il-6* by comparing the respective sequence with similarities of binding matrices from the database. Briefly, using this software tool, an index was calculated, representing the essential binding regions for the respective binding site. A matrix was defined as a selective description of DNA patterns to bind transcription factors attributed to a database. The matrix similarity evaluated the matches of all necessary bases of the binding sequence of a transcription factor to the region of interest and was considered relevant for values ≥0.8. Each matrix contained a core sequence which was defined as the highest conserved positions of the matrix (usually 4 bp) reflected by the core similarity with a maximum score of 1.0.

### 4.2. Cell Preparation and Culture of CD4^+^CD25^+^

CD4^+^CD25^+^ (nTregs) and CD4^+^CD25^−^ T cells from spleens of naive C57BL/6 (WT) and CD8-deficient (CD8^−/−^) mice were isolated by collagenase digestion and enriched using nylon wool columns as described previously [13]. Lymphocytes were further purified by CD4^+^CD25^+^ regulatory T cell MACS beads (Miltenyi Biotec, Bergisch-Gladbach, Germany), resulting in a purity of >95% of CD4^+^CD25^+^ cells. Cells were washed, counted, and resuspended to a final concentration of 14 × 10^6^ cells per mL in complete RPMI 1640 (Mediatech Cellgro, Manassas, VA, USA) tissue culture medium, containing heat-inactivated fetal calf serum (FCS 10%; Sigma-Aldrich, St. Louis, MO, USA), L-glutamine (5 mM), β-mercaptoethanol (2 mM), hepes buffer (15 mM), penicillin (100 U/mL), and streptomycin (100 μg/mL) (all from Gibco, Grand Island, NY). Cells were cultured in media or in the presence of PMA (50 ng/mL) and ionomycin (1 μM, both Sigma-Aldrich, St. Louis, MO, USA) for 3 h.

### 4.3. Electrophoretic Mobility Shift Assay (EMSA)

Nuclear extracts were prepared [23] from cultured nTregs from WT or CD8^−/−^ mice (14 × 10^6^ cells) in four independent experiments. Cells were resuspended in 120 µL of Buffer A (10 mM 4-(2-hydroxyethyl)-1-piperazineethanesulfonic acid (HEPES), 3 mM MgCl_2_, 40 mM KCl, 1 mM dithiothreitol (DTT), 5% glycerol, 0.2% NP-40) supplemented with protease (1 mM phenylmethanesulfonylfluoride (PMSF), 10 µg/mL aprotinin, 10 µg/mL leupeptin, 10 µg/mL antipain, 10 µg/mL pepstatin) and phosphatase inhibitors (1 mM benzamidine, 1 mM orthosodium vanadate, 1 mM sodium fluoride, 5 mM β-glycerophosphate) [66]. Sedimented nuclei were re-suspended in Buffer C (20 mM HEPES, 1.5 mM MgCl_2_, 420 mM NaCl, 0.2 mM ethylenediaminetetraacetic acid (EDTA), 1 mM DTT, 25% glycerol, supplemented with protease and phosphatase inhibitors as in Buffer A) in corresponding volumes. Nuclear extracts were aliquoted and stored at −80 °C. The protein concentration of nuclear extracts was determined using the bicinchoninic acid (BCA) kit (Thermo Fisher Scientific, Waltham, MA). Annealing of the complementary oligonucleotide pairs was carried out in 10 mM Tris Cl (pH 8), 1 mM EDTA and 100 mM NaCl by boiling the samples for 5 min and cooling slowly to 37 °C. Purified double stranded probes (100 ng) were end-labeled using g [^32^P]-ATP (250 µCi) with T4 polynucleotide kinase (New England Biolabs, Ipswich, MA). Free radioactivity was removed by mini Quick Spin Oligo Columns (Roche Mannheim, Germany). For EMSA experiments, each binding reaction (20 µL) contained 5 µg nuclear extract, 1x binding buffer (10 mM Tris Cl pH 8, 1 mM EDTA, 0.1 mM β-mercaptoethanol), 0.25 µg/reaction Poly(dI-dC)-Poly(dI-dC) and was adjusted for a final concentration of 80 mM NaCl and 4% glycerol. Supershift experiments for the transcription factors, NF-kB (p50 (sc-1190X), p65 (sc-109X)), SP1, 2, 3, and 4 (sc-14027X, sc-17814X, sc-644X, sc-645X), c-Fos (sc-253X), c-Jun (sc-44X), USF1 (sc-229X), USF2 (sc-862X) and C/EBP (sc-151X, sc-746X) (all Santa Cruz, Santa Cruz, CA) were performed by pre-incubation with antibody (4 µg) for 30 min on ice. The respective ^32^P-labeled protein/DNA complexes were resolved on a 5% polyacrylamide gel. Competition experiments were performed to verify the specificity of the observed complexes by pre-incubating the nuclear extract with a 100-fold molar excess of a competing unlabeled oligonucleotide as indicated prior to the addition of the labeled probe. Consensus sequences for the transcription factors, NF-kB [23,24], SP [23,27], AP1 [26], and USF [27,48] were also used for competition experiments (100-fold molar excess). All oligonucleotide sequences are presented in the Online Supplemental Table S2. EMSAs for each of the *Il-6* regions of interest were repeated independently using different nuclear extracts from nTregs of both mouse strains. Representative EMSAs are included in the manuscript.

### 4.4. IL-6 Reporter Constructs

DNA was isolated from nTregs from WT or CD8^−/−^ mice. To study the promoter activity of *Il-6*, 1138 bp upstream of the translation start site of *Il-6* (Online Supplemental Figure S2) was cloned into the pGL4.10 basic vector (Promega, Madison, WI) using the Gibson Assembly Cloning kit according to manufacturer’s protocol (Thermo Fisher Scientific, Waltham, MA, USA). A total of 51 colonies for WT and 53 for the CD8^−/−^ strain were screened by Sanger sequencing to delineate the distribution of a GT STR in the *Il-6* promoter. Following the manufacturer’s protocol, site-directed mutagenesis (Agilent Technologies, Santa Clara, CA, USA) of the pGL4 *Il-6* construct was performed to deplete potential transcription factor binding sites in the *Il-6* promoter region. The sequence was confirmed by re-sequencing of all *Il-6* promoter constructs. Primers for amplification of the *Il-6* promoter region, for Sanger sequencing, and for the site-directed mutagenesis are depicted in the Online Supplemental Table S3.

### 4.5. Luciferase Assay

NIH3T3 mouse fibroblast cells were seeded in 96-well plates at a density of 7 × 10^4^ per well. The next day, cells were transfected with 750 ng pGL4.10 plasmids (Promega, Madison, WI, USA) expressing the luciferase gene under the control of the *Il-6* promoter containing the respective wild-type, point mutation at one of the four analyzed transcription factor binding sites, or different lengths of the GT repeat clones together with 10 ng of the pRL-TK *Renilla* reporter plasmid (Promega, Madison, WI, USA) for normalization of transfection efficiency and cell viability. Lipofectamine 2000 was used as the transfection reagent according to the manufacturer’s protocol (Thermo Fisher Scientific, Waltham, MA, USA). Directly after transfection, the medium was exchanged with a medium containing pure medium (unstimulated) or 10 ng/mL LPS, 0.5 ng/mL TNFα, 1 ng/mL TNFα. Fourteen hours after stimulation, cells were washed in PBS and lysed in 1x passive lysis buffer (Promega, Madison, WI, USA). Dual luciferase assay was performed according to manufacturer’s protocol (Promega, Madison, WI, USA). Luciferase and renilla signals were measured using a Synergy HT luminometer (BioTek, Winooski, VT, USA) and quantified as relative light units (RLU). Experiments were conducted independently four to five times with two technical replicates for each construct. A paired, two-tailed Student’s t-test was performed to identify significant differences between groups (*p* ≤ 0.05).

## 5. Conclusions

Changes in transcription factor binding and a novel variable dinucleotide repeat of variable length impact the regulation of *Il-6* contributing to nTreg plasticity important for IL-6-related disease with impaired nTreg function.

## Data Availability

The data supporting the findings of this study are available from the corresponding author upon request.

## Supplementary Materials

The following are available online at https://www.mdpi.com/article/10.3390/ijms22094582/s1.

## Abbreviations

BCA	bicinchoninic acid
DTT	dithiothreitol
E-box	enhancer box
EDTA	ethylenediaminetetraacetic acid
EMSA	electrophoretic mobility shift assay
FEV1	forced expiratory volume in 1 s
Foxp3	forkhead-box-protein p3
GITR	glucocorticoid-induced TNFR family related protein
HEPES	4-(2-hydroxyethyl)-1-piperazineethanesulfonic acid
JNK	c-Jun N-terminal kinase
LPS	lipopolysaccharide
ORMDL3	ORMDL sphingolipid biosynthesis regulator 3
PMA	phorbol 12-myristate 13-acetate
PMSF	phenylmethanesulfonylfluoride
RLU	relative light units
sIL-6R	soluable IL-6 receptor
SP	specificity protein
STR	short tandem repeat
TGF-β	tumor growth factor β
TNFα	tumor necrosis factor α
nTreg	naturally occurring CD4^+^CD25^+^Foxp3^+^ T regulatory cell
Treg	T regulatory cell
TSS	transcription start site
USF	upstream stimulatory factor
WT	wild-type
VISTA	visualization tool for alignment
