# Supplementary material for: Plasticity of Naturally Occurring Regulatory T Cells in Allergic Airway Disease Is Modulated by the Transcriptional Activity of Il-6
Source: Int J Mol Sci. 2021 Apr 27;22(9):4582. doi: 10.3390/ijms22094582 (PMC8123826; doi:10.3390/ijms22094582)
Supplement: Supplementary file 1 [file ijms-22-04582-s001.zip › ijms-1174895-supplementary.pdf]

Plasticity of Naturally Occurring Regulatory T Cells in Allergic Airway Disease is Modulated by the Transcriptional Activity of *Il-6*

Morgan MacBeth [1,2], Anthony Joetham [1], Erwin W. Gelfand [1] and Michaela Schedel [1,3,4,*]

1. Division of Allergy and Immunology and Cell Biology, Department of Pediatrics, National Jewish Health, Denver, CO 80206, USA; morgan.macbeth@cuanschutz.edu (M.M.); joethama@njhealth.org (A.J.), erwin3460@gmail.com (E.W.G.)
2. Department of Medical Oncology, University of Colorado, Denver, CO 80206, USA
3. Department of Pulmonary Medicine, University Medical Center Essen-Ruhrlandklinik, 45239 Essen, Germany; michaela.schedel@rlk.uk-essen.de (M.S.)
4. University Hospital Essen, University Duisburg-Essen, 45147 Essen, Germany

* Correspondence: michaela.schedel@rlk.uk-essen.de; Tel.: +49-201-723-82545

Citation: MacBeth, M.; Joetham, A.; Gelfand, E.W.; Schedel, M. Plasticity of Naturally Occurring Regulatory T Cells in Allergic Airway Disease Is Modulated by the Transcriptional Activity of Il-6. Int. J. Mol. Sci. 2021, 22, 4582. https://doi.org/10.3390/ijms22094582

Academic Editor: Daniel P. Potaczek

Received: 23 March 2021
Accepted: 26 April 2021
Published: 27 April 2021

Publisher's Note: MDPI stays neutral with regard to jurisdictional claims in published maps and institutional affiliations.

Copyright: © 2021 by the authors. Licensee MDPI, Basel, Switzerland. This article is an open access article distributed under the terms and conditions of the Creative Commons Attribution (CC BY) license (http://creativecommons.org/licenses/by/4.0/).

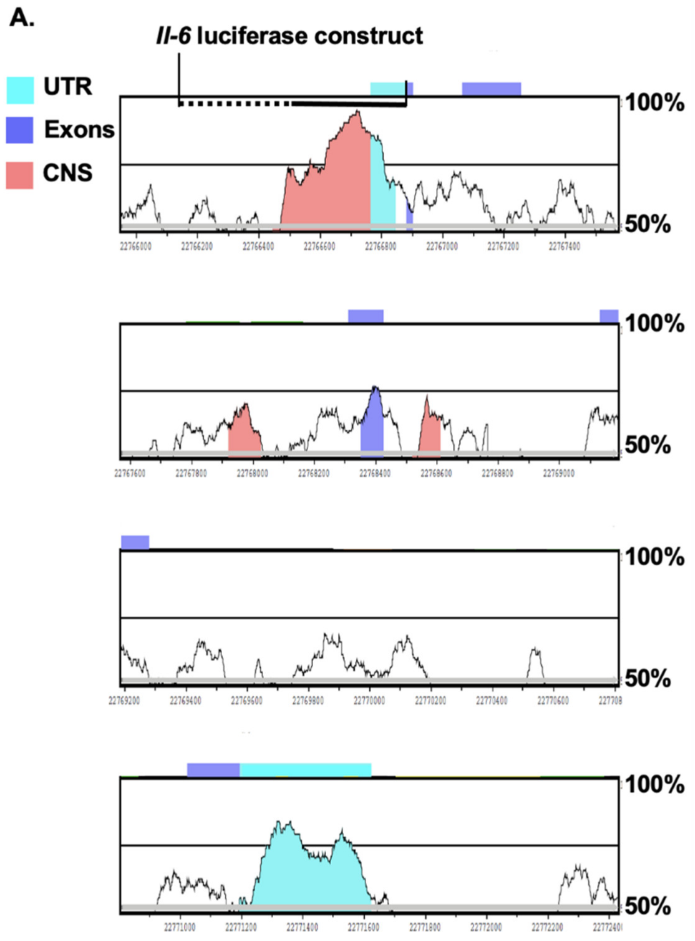

Online Supplemental Figure 1A. The promoter regions of the human *IL-6* and mouse *Il-6* promoter share high homology. Percentage of alignment between the human *IL-6* and mouse *Il-6* gene using the VISTA genome browser software. Location of the luciferase construct (dotted and solid line) including the region of sequence homology in humans and mice as shown in Online Supplemental Figure 1B (solid line) are

depicted. Red = conserved non-coding sequence (CNS), turquoise = untranslated region (UTR), blue = exons.

B.

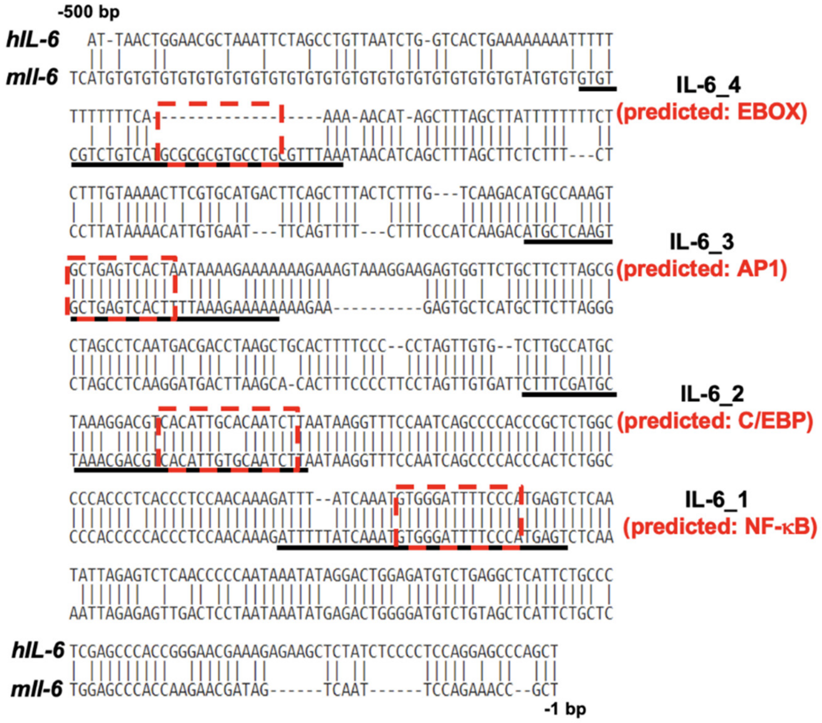

Online Supplemental Figure 1B. Sequence homology between the human (upper panel) and mouse (lower panel) *IL-6* promoter region. The sequence of both species (500 bp upstream of the translation start site) are shown including the four analyzed regions by electrophoretic mobility shift assay, predicted transcription factor binding to each of the studied *Il-6* loci, and the GT repeat. Relative distance to the translation start site was included.

```
-1139 AGACTTGAGC ATTGGAGGGG TTATTCAGAG TGAGACGTAC CACCTTCAGA TTCAAATCCT GTCATCCAGT AGAAGGGAGC TTCAAACACA AGCTAGCTAA
      TCTGAACTCG TAACCTCCCC AATAAGTCTC ACTCTGCATG GTGGAAGTCT AAGTTTAGGA CAGTAGGTCA TCTTCCCTCG AAGTTTGTGT TCGATCGATT
          luciferase construct 5' start
-1039 GATACAATGA GGTCCTTCTT CGATATCTTT ATCTTCCATA TACCATGAAT CAAAGAAACT TCAACAACAT GAGGACTGCA ACAGACCTTC AAGCCTCCTT
      CTATGTTACT CCAGGAAGAA GCTATAGAAA TAGAAGGTAT ATGGTACTTA GTTTCTTTGA AGTTGTTGTA CTCCTGACGT TGTCTGGAAG TTCGGAGGAA

 -939 GCATGACCTG GAAATGTTTT GGGGTGTCCT GGCAGCAGTG GGATCAGCAC TAACAGATAA GGGCAACTCT CACAGAGACT AAAGGTCTTA ACTAAGAAGA
      CGTACTGGAC CTTTACAAAA CCCCACAGGA CCGTCGTCAC CCTAGTCGTG ATTGTCTATT CCCGTTGAGA GTGTCTCTGA TTTCCAGAAT TGATTCTTCT

 -839 TAGCCAAGAG ACCACTGGGG AGAATGCAGA GAATAGGCTT GGACTTGGAA GCCAAGATTG CTTGACAACA GACAGAAGAT ATTTCTGTAC TTCACCCACT
      ATCGGTTCTC TGGTGACCCC TCTTACGTCT CTTATCCGAA CCTGAACCTT CGGTTCTAAC GAACTGTTGT CTGTCTTCTA TAAAGACATG AAGTGGGTGA

 -739 TTACCCACCT GGCAACTCCT GGAAACAACT GCACAAAATT TGGAGGTGAA CAAACCATTA GAAACAACTG GTCCTGACAA GACACAGGAA AAACAAGCAA
      AATGGGTGGA CCGTTGAGGA CCTTTGTTGA CGTGTTTTAA ACCTCCACTT GTTTGGTAAT CTTTGTTGAC CAGGACTGTT CTGTGTCCTT TTTGTTCGTT

 -639 TATGCAACAT TACTGTCTGT TGTCCAGGTT GGGTGCTGGG GGTGGGAGAG GGAGTGTGTG TCTTTGTATG ATCTGAAAAA ACTCAGGTCA GAACATCTGT
      ATACGTTGTA ATGACAGACA ACAGGTCCAA CCCACGACCC CCACCCTCTC CCTCACACAC AGAAACATAC TAGACTTTTT TGAGTCCAGT CTTGTAGACA
                                                                          GT-repeat
 -539 AGATCCTTAC AGACATACAA AAGAATCCTA GCCTCTTATT CATGTGTGTG TGTGTGTGTG TGTGTGTGTG TGTGTGTGTG TGTGTGTGTA TGTGTGTGTC
      TCTAGGAATG TCTGTATGTT TTCTTAGGAT CGGAGAATAA GTACACACAC ACACACACAC ACACACACAC ACACACACAC ACACACACAT ACACACACAG
            Il-6_4 (predicted: EBOX)
 -439 GTCTGTCATG CGCGCGTGCC TGCGTTTAAA TAACATCAGC TTTAGCTTCT CTTTCTCCTT ATAAAACATT GTGAATTTCA GTTTTCTTTC CCATCAAGAC
      CAGACAGTAC GCGCGCACGG ACGCAAATTT ATTGTAGTCG AAATCGAAGA GAAAGAGGAA TATTTTGTAA CACTTAAAGT CAAAAGAAAG GGTAGTTCTG

                 Il-6_3 (predicted: AP1)
 -339 ATGCTCAAGT GCTGAGTCAC TTTTAAAGAA AAAAAAGAAG AGTGCTCATG CTTCTTAGGG CTAGCCTCAA GGATGACTTA AGCACACTTT CCCCTTCCTA
      TACGAGTTCA CGACTCAGTG AAAATTTCTT TTTTTTCTTC TCACGAGTAC GAAGAATCCC GATCGGAGTT CCTACTGAAT TCGTGTGAAA GGGGAAGGAT
                                                                                              transcription start site
                     Il-6_2 (predicted: C/EBP)
 -239 GTTGTGATTC TTTCGATGCT AAACGACGTC ACATTGTGCA ATCTTAATAA GGTTTCCAAT CAGCCCCACC CACTCTGGCC CCACCCCCAC CCTCCAACAA
      CAACACTAAG AAAGCTACGA TTTGCTGCAG TGTAACACGT TAGAATTATT CCAAAGGTTA GTCGGGGTGG GTGAGACCGG GGTGGGGGTG GGAGGTTGTT

            Il-6_1 (predicted: NF-κB)
 -139 AGATTTTTAT CAAATGTGGG ATTTTCCCAT GAGTCTCAAA ATTAGAGAGT TGACTCCTAA TAAATATGAG ACTGGGGATG TCTGTAGCTC ATTCTGCTCT
      TCTAAAAATA GTTTACACCC TAAAAGGGTA CTCAGAGTTT TAATCTCTCA ACTGAGGATT ATTTATACTC TGACCCCTAC AGACATCGAG TAAGACGAGA

                                          translation start site
  -39 GGAGCCCACC AAGAACGATA GTCAATTCCA GAAACCGCTA TGAAGTTCCT CTCTGCAAGT AAGTGAAGGC AGTTCCTTGC CCTCTGGCGG AGCTATTGAG
      CCTCGGGTGG TTCTTGCTAT CAGTTAAGGT CTTTGGCGAT ACTTCAAGGA GAGACGTTCA TTCACTTCCG TCAAGGAACG GGAGACCGCC TCGATAACTC
                  luciferase construct 3' end
```

Online Supplemental Figure 2. Sequence of the mouse *Il-6* promoter region. The four analyzed regions by electrophoretic mobility shift assay and the GT repeat are underlined. The location of the cloned *Il-6* promoter region is depicted. Transcription and translation start site of *Il-6* are marked. Relative distance to the translation start site is shown.

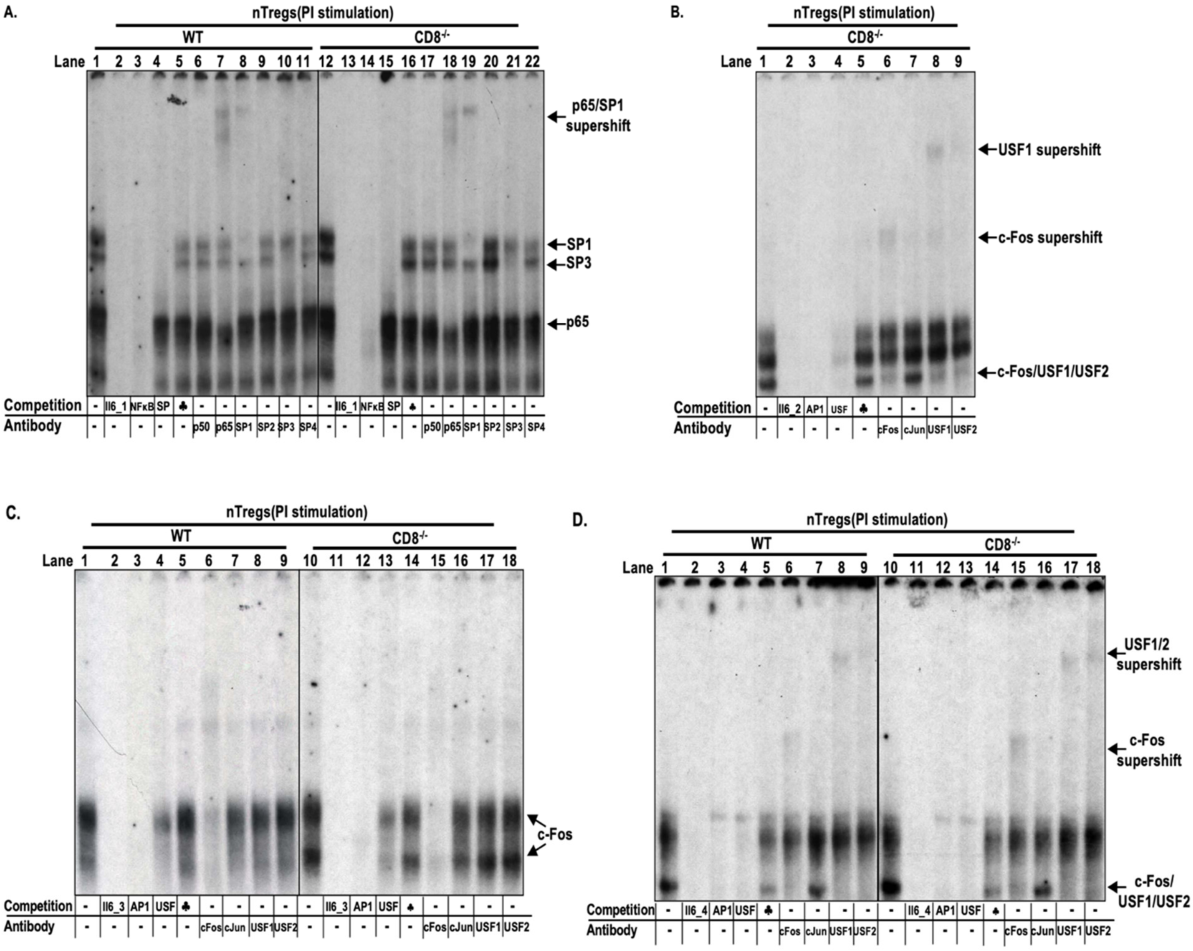

Online Supplemental Figure 3. Distinct affinity of transcription factor binding to promoter regions within the *Il-6* gene in nTregs from wild-type (WT) and CD8$^{-/-}$ mice. A-D. EMSA analyses of four different *Il-6* promoter loci upstream of the transcription start site of the *Il-6* gene were performed including **A.** Il-6_1, **B.** Il-6_2, **C.** Il-6_3, and **D.** Il-6_4 using nuclear extract isolated from nTregs of WT or CD8$^{-/-}$ (KO) mice (5 μg) stimulated with PMA/ionomycin (50 ng/ml/1 μM) for 3 hours. Competitors (100-fold molar excess) and supershift antibodies (4 μg) for each experiment are noted below the respective lanes. ♣: competition experiment using an unrelated oligo.

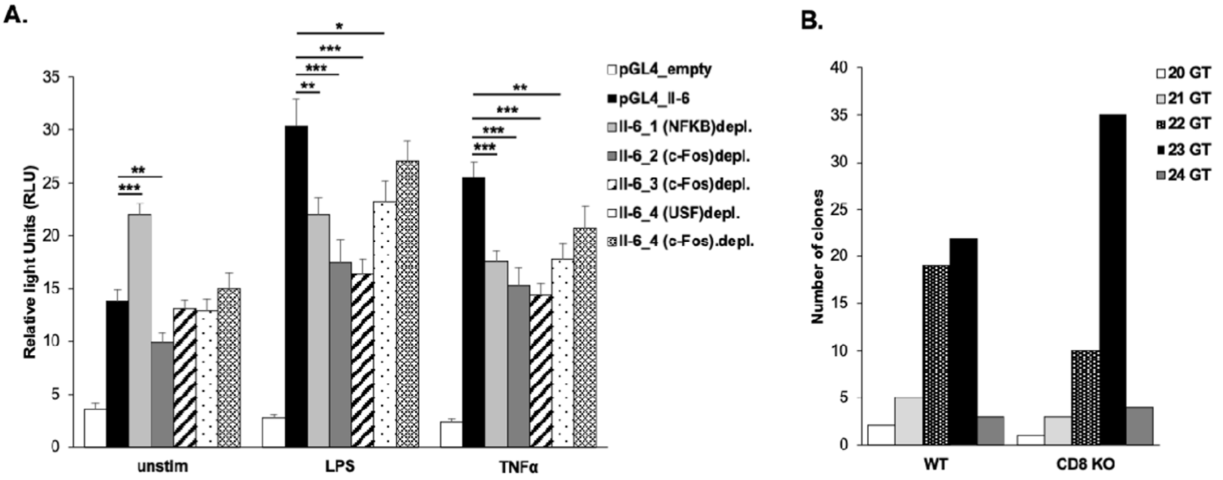

Online Supplemental Figure 4. Four defined regions are essential for transcription factor binding and a novel variable length STR is found in the *Il-6* promoter A. NIH3T3 cells were transiently transfected with the empty pGL4 luciferase vector (pGL4 empty) or with different reporter constructs of the *Il-6* promoter (1,138 bp). In the construct Il-6_1 (NF-κB)depl., the NF-κB binding site as identified by EMSA within Il-6_1 was depleted by site-directed mutagenesis. Correspondingly, the c-Fos binding site within Il-6_2 [Il-6_2(c-Fos)depl.], the c-Fos binding site within Il-6_3 [Il-6_3(c-Fos)depl.], the USF binding site within Il-6_4 [Il-6_4(USF)depl.], and the c-Fos binding site within Il-6_4 [Il-6_4(c-Fos)depl.] were mutated (4-5 independent experiments, 2 technical replicates). Cells were left in medium or stimulated with 10 ng/ml LPS or 0.5 ng/ml TNFα. Luciferase activity was normalized for transfection efficiency using the control plasmid pRL-TK. The relative luciferase activity is presented in relative light units (RLU). Paired, two tailed students t-test was performed. *p≤0.05, **p≤0.01, ***p≤0.001. **B.** *Il-6* luciferase promoter constructs were generated using DNA isolated from wild-type (WT) or $CD8^{-/-}$ (CD8 KO) nTregs. To determine the distribution of a GT short tandem repeat in nTregs of both strains, a total of 104 clones (WT=51, $CD8^{-/-}$=53) were sequenced. Distribution of number of clones in nTregs from each strain containing 20-24 GT repeats are depicted.

Supplemental Table 1: *In silico* transcription factor (TF) binding analyses of the *Il-6* promoter region including 600 bp upstream of the translation start site. Relative distance upstream of the translation start site and the chromosomal strand (+ or -) of the predicted binding of the TF are depicted. Capital letters in the sequence indicate the core sequence of a predicted TF binding matrix with the highest degree of conservation. Letters marked in red reflect the conserved sequences (>60%) between the TF matrix and *Il-6*. Marked in bold are predicted TF binding regions further evaluated by electrophoretic mobility shift assay.

TF	TF family	Name of TF family	Relative distance to TLSS (bp)		Strand	Core similarity	Matrix similarity	Sequence
BTEB3	*KLFS*	Krueppel like transcription factors	-595	-579	+	1.000	0.939	ggagagGGAGtgtgtgt
GAGA	*GABF*	GA-boxes	-598	-574	+	1.000	0.81	gtgggAGAGggagtgtgtgtctttg
TIEG	*SP1F*	GC-Box factors SP1/GC	-591	-577	+	0.750	0.879	agGGAGtgtgtgtct
TAXCREB	*CREB*	cAMP-responsive element binding proteins	-591	-571	-	0.750	0.713	atacaaAGACacacactccct
GATA3	*GATA*	GATA binding factors	-575	-563	-	1.000	0.932	ttcAGATcataca
AREB6	*ZFHX*	Two-handed zinc finger homeodomain transcription factors	-559	-547	-	1.000	0.945	ttctgACCTgagt
REV-ERBA	*RORA*	v-ERB and RAR-related orphan receptor alpha	-563	-541	+	1.000	0.895	aaaaactcagGTCAgaacatctg
FHXB	*FKHD*	Fork head domain factors	-556	-540	+	0.818	0.853	caggtcAGAAcatctgt
TAL1ALPHAE47	*HAND*	Twist subfamily of class B bHLH transcription factors	-554	-534	-	1.000	0.952	ggatctaCAGAtgttctgacc
NGN_NEUROD	*NEUR*	NeuroD, Beta2, HLH domain	-550	-538	+	1.000	0.985	agaaCATCtgtag
RP58	*RP58*	RP58 (ZFP238) zinc finger protein	-550	-538	+	1.000	0.954	agaaCATCtgtag
PRE	*GREF*	Glucocorticoid responsive and related elements	-552	-534	-	1.000	0.851	ggatctacagaTGTTctga
POU3F3	*OCT1*	Octamer binding protein	-510	-494	-	0.784	0.823	cacatGAATaagaggct
PIT1	*PIT1*	GHF-1 pituitary specific pou domain transcription factor	-509	-495	+	1.000	0.93	gcctcTTATtcatgt
PAX4_PD	*PAX6*	PAX-4/PAX-6 paired domain binding sites	-436	-418	-	1.000	0.919	cagGCACgcgcgcatgaca
ZF5	*ZF5F*	ZF5 POZ domain zinc finger	-432	-422	+	1.000	0.967	atgcGCGCgtg
HELT	*HESF*	Vertebrate homologues of enhancer of split complex	-432	-418	-	1.000	0.959	caggCACGcgcgcat
AHRARNT	*AHRR*	AHR-arnt heterodimers and AHR-related factors	-436	-412	+	1.000	0.952	tgtcatgcgcGCGTgcctgcgttta
MYCMAX	***EBOX***	**E-box binding factors**	**-430**	**-418**	**-**	**1.000**	**0.921**	**caggcaCGCGcgc**
OTP	*CART*	Cart-1 (cartilage homeoprotein 1)	-423	-403	-	0.751	0.841	gatgttATTTaaacgcaggca

E4BP4	*CREB*	cAMP-responsive element binding proteins	-421	-401	-	0.758	0.852	ctgatgttatTTAAacgcagg
TEF_HLF	*PARF*	PAR/bZIP family	-419	-403	+	0.784	0.82	tgcgtTTAAataacatc
E4BP4	*CREB*	cAMP-responsive element binding proteins	-420	-400	+	0.769	0.803	ctgcgtttaaATAAcatcagc
LHX3	*LHXF*	Lim homeodomain factors	-421	-399	-	0.751	0.812	agctgatgttATTTaaacgcagg
TEF	*PARF*	PAR/bZIP family	-418	-402	-	0.773	0.888	tgatgttatTTAAacgc
MTBF	*HMTB*	Human muscle-specific Mt binding site	-413	-405	-	1.000	0.902	tgttATTTa
PROP1	*CART*	Cart-1 (cartilage homeoprotein 1)	-418	-398	+	0.753	0.844	gcgtttAAATaacatcagctt
CREB	*CREB*	cAMP-responsive element binding proteins	-416	-396	-	0.862	0.863	taaagcTGATgttatttaaac
BARBIE	*BARB*	Barbiturate-inducible element box from pro+eukaryotic genes	-407	-393	-	1.000	0.91	agctAAAGctgatgt
EVI1	*EVI1*	EVI1-myleoid transforming protein	-403	-387	-	0.750	0.841	aagagAAGCtaaagctg
IRF7	*IRFF*	Interferon regulatory factors	-401	-381	-	1.000	0.892	aggaGAAAgagaagctaaagc
LTATA	*VTBP*	Vertebrate TATA binding protein factor	-390	-374	-	1.000	0.84	tttTATAaggagaaaga
HOXD13	*ABDB*	Abdominal-B type homeodomain transcription factors	-386	-370	+	1.000	0.919	tctccttaTAAAacatt
CDX2	*CDXF*	Vertebrate caudal related homeodomain protein	-386	-368	-	1.000	0.852	acaatgtTTTAtaaggaga
PBX_HOXA9	*HOXC*	HOX - PBX complexes	-384	-368	-	0.750	0.849	acaaTGTTttataagga
VTATA	*VTBP*	Vertebrate TATA binding protein factor	-383	-367	+	1.000	0.941	ccttaTAAAacattgtg
FHXB	*FKHD*	Fork head domain factors	-378	-362	-	0.909	0.855	aaattcACAAtgtttta
FAST1	*FAST*	FAST-1 SMAD interacting proteins	-375	-359	+	1.000	0.884	aacatTGTGaatttcag
IRF1	*IRFF*	Interferon regulatory factors	-371	-351	-	1.000	0.901	aaagaaaactGAAAttcacaa
HMGIY	*SORY*	SOX/SRY-sex/testis determinig and related HMG box factors	-372	-350	+	1.000	0.93	attgtgAATTtcagttttctttc
DINR	*INRE*	Core promoter initiator elements	-364	-354	+	1.000	0.965	ttTCAGtttc
RBPJK	*RBPF*	RBPJ - kappa	-357	-343	-	1.000	0.946	ttgaTGGGaaagaaa
PAX1	*PAX1*	PAX-1 binding sites	-355	-337	+	0.750	0.689	tCTTTcccatcaagacatg
OCT3_4	*OCT1*	Octamer binding protein	-342	-326	+	1.000	0.824	gacATGCtcaagtgctg
P53	*P53F*	p53 tumor suppressor	-344	-322	-	1.000	0.935	gactcagcacttgagCATGtctt
DEC2	*HESF*	Vertebrate homologues of enhancer of split complex	-339	-325	-	0.871	0.972	tcagcaCTTGagcat
NKX25	*NKXH*	NKX homeodomain factors	-339	-321	+	1.000	1	atgctcAAGTgctgagtca

TCF11MAFG	AP1R	MAF and AP1 related factors	-335	-315	-	1.000	0.957	taaaagTGACtcagcacttga
**AP1**	**AP1F**	**AP1, Activating protein 1**	**-329**	**-319**	**+**	**1.000**	**0.968**	**gctgAGTCAct**
**AP1**	**AP1F**	**AP1, Activating protein 1**	**-329**	**-319**	**-**	**0.971**	**0.974**	**agTGACtcagc**
NFE2	AP1R	MAF and AP1 related factors	-333	-313	+	1.000	1	aagtgCTGAgtcacttttaaa
VTATA	VTBP	Vertebrate TATA binding protein factor	-326	-310	-	1.000	0.927	ttctTAAAagtgactc
BARBIE	BARB	Barbiturate-inducible element box from pro+eukaryotic genes	-310	-296	+	1.000	0.888	aaaaAAAGaagagtg
EVI1.07	EVI1	EVI1-myleoid transforming protein	-310	-294	+	1.000	0.916	aaaaaAAGAagagtgct
PAX2	PAX8	PAX-2/5/8 binding sites	-297	-283	+	0.923	0.94	tgCTCAtgcttctta
HMX2	HOMF	Homeodomain transcription factors	-272	-254	+	1.000	0.916	caaggatgaCTTAagcaca
CREB2	CREB	cAMP-responsive element binding proteins	-272	-252	+	1.000	0.906	caaggaTGACttaagcacact
ATATA	VTBP	Vertebrate TATA binding protein factor	-269	-253	-	1.000	0.812	gtgtgctTAAGtcatcc
HMX2	HOMF	Homeodomain transcription factors	-267	-249	-	1.000	0.896	gaaagtgtgCTTAagtcat
MYT1	MYT1	MYT1 C2HC zinc finger protein	-260	-248	-	1.000	0.799	ggaAAGTgtgctt
BLIMP1	PRDF	Positive regulatory domain I binding factor	-261	-243	-	1.000	0.838	gaagggGAAAgtgtgctta
IRF7	IRFF	Interferon regulatory factors	-259	-239	-	0.821	0.905	ctagGAAGgggaaagtgtgct
MZF1	MZF1	Myeloid zinc finger 1 factors	-254	-244	-	1.000	1	aaGGGGaaagt
GABP	ETSF	Human and murine ETS1 factors	-254	-234	-	1.000	0.868	cacaactaGGAAggggaaagt
IRF4	IRFF	Interferon regulatory factors	-236	-216	-	1.000	0.942	gtttagcatcGAAAgaatcac
PAX2	PAX2	PAX-2 binding sites	-236	-214	+	1.000	0.788	gtgattctttcgatgctAAACga
CREB1	CREB	cAMP-responsive element binding proteins	-223	-203	-	1.000	0.974	acaatgTGACgtcgtttagca
E4F	E4FF	Ubiquitous GLI - Krueppel like zinc finger involved in cell cycle regulation	-217	-205	+	1.000	0.893	acgACGTcacatt
CREB	CREB	cAMP-responsive element binding proteins	-220	-200	-	1.000	0.902	tgcacaatgTGACgtcgttta
SOX9	SORY	SOX/SRY-sex/testis determinig and related HMG box factors	-218	-196	-	1.000	0.944	agattgcACAAtgtgacgtcgtt
**CEBPB**	**CEBP**	**Ccaat/Enhancer Binding Protein**	**-210**	**-196**	**+**	**1.000**	**0.989**	**cacattgtGCAAtct**

HLF	*PARF*	PAR/bZIP family	-211	-195	+	0.836	0.869	tcacattgtGCAAtctt
HHEX	*HOMF*	Homeodomain transcription factors	-206	-188	+	1.000	0.953	ttgtgcaatctTAATaagg
BRN3	*BRNF*	Brn POU domain factors	-202	-184	+	1.000	0.84	gcaatctTAATaaggtttc
VAX2	*HBOX*	Homeobox transcription factors	-202	-184	-	1.000	0.852	gaaaccttATTAagattgc
BARX2	*HOMF*	Homeodomain transcription factors	-202	-184	+	1.000	0.952	gcaatctTAATaaggtttc
NKX61	*NKX6*	NK6 homeobox transcription factors	-200	-186	+	1.000	0.915	aatcTTAAtaaggtt
BRN5	*BRN5*	Brn-5 POU domain factors	-203	-181	-	1.000	0.922	ttggaaaccttATTAagattgca
OCT1	*OCT1*	Octamer binding protein	-200	-184	-	1.000	0.861	gaaaccttATTAagatt
NKX25	*NKXH*	NKX homeodomain factors	-200	-182	+	1.000	0.899	aatctTAATaaggtttcca
NFAT5	*NFAT*	Nuclear factor of activated T-cells	-198	-180	-	1.000	0.888	attGGAAaccttattaaga
BRN2	*BRNF*	Brn POU domain factors	-195	-177	-	0.900	0.879	ctGATTggaaaccttatta
NFY	*CAAT*	CCAAT binding factors	-188	-174	+	1.000	0.951	gtttCCAAtcagccc
CDP	*CLOX*	CLOX and CLOX homology (CDP) factors	-189	-171	+	1.000	0.969	ggtttcCAATcagccccac
INSM1	*INSM*	Insulinoma associated factors	-180	-168	-	1.000	0.907	tgggtGGGGctga
BKLF	*KLFS*	Krueppel like transcription factors	-182	-166	-	1.000	0.99	agtGGGTggggctgatt
MAZR	*MAZF*	Myc associated zinc fingers	-179	-167	-	1.000	0.895	gtgggtGGGGctg
GC	*SP1F*	GC-Box factors SP1/GC	-180	-166	-	0.872	0.942	agtgGGTGgggctga
WT1	*EGRF*	EGR/nerve growth factor induced protein C & related factors	-179	-163	-	1.000	0.932	cagagTGGGtggggctg
GLI3	*GLIF*	GLI zinc finger family	-178	-164	+	1.000	0.881	agccCCACccactct
NGFIC	*EGRF*	EGR/nerve growth factor induced protein C & related factors	-177	-161	-	0.770	0.833	gccaGAGTgggtggggc
PLAG1	*PLAG*	Pleomorphic adenoma gene	-176	-156	-	0.833	0.881	GTGGggccagagtgggtgggg
BKLF	*KLFS*	Krueppel like transcription factors	-167	-151	-	1.000	0.998	tggGGGTggggccagag
MAZR	*MAZF*	Myc associated zinc fingers	-164	-152	-	1.000	0.949	gggggtGGGGcca
GC	*SP1F*	GC-Box factors SP1/GC	-165	-151	-	0.872	0.945	tgggGGTGgggccag
GKLF	*KLFS*	Krueppel like transcription factors	-165	-149	-	1.000	0.984	ggtggggGTGGggccag
PLAG1	*PLAG*	Pleomorphic adenoma gene	-166	-146	-	1.000	0.885	GAGGgtgggggtggggccaga
ZBP89	*ZBPF*	Zinc binding protein factors	-166	-144	+	1.000	0.976	tctggccccaCCCCcaccctcca
WT1	*EGRF*	EGR/nerve growth factor induced protein C & related factors	-162	-146	-	1.000	0.998	gagggTGGGggtggggc

RREB1	*RREB*	Ras-responsive element binding protein	-161	-147	+	1.000	0.876	cCCCAcccccaccct
BKLF	*KLFS*	Krueppel like transcription factors	-161	-145	-	1.000	0.974	ggaGGGTgggggtgggg
GC	*SP1F*	GC-Box factors SP1/GC	-159	-145	-	0.872	0.893	ggagGGTGggggtgg
ZNF219	*ZBPF*	Zinc binding protein factors	-163	-141	+	1.000	0.941	ggccccaCCCCcaccctccaaca
SPZ1	*SPZ1*	Testis-specific bHLH-Zip transcription factors	-154	-144	-	1.000	0.958	tGGAGggtggg
LEF1	*LEFF*	LEF1/TCF	-149	-133	+	1.000	0.876	cctccaaCAAAgatttt
GFI1	*GFI1*	Growth factor independence transcriptional repressor	-146	-132	-	1.000	0.905	aaaAATCtttgttgg
SOX2	*SORY*	SOX/SRY-sex/testis determinig and related HMG box factors	-150	-128	+	1.000	0.929	ccctccaACAAagatttttatca
CDX2	*CDXF*	Vertebrate caudal related homeodomain protein	-141	-123	+	1.000	0.862	aaagattTTTAtcaaatgt
GATA	*GATA*	GATA binding factors	-137	-125	-	1.000	0.936	atttGATAaaaat
SMARCA3	*RUSH*	SWI/SNF related nucleophosphoproteins with a RING finger DNA binding motif	-131	-121	-	0.960	0.965	ccACATttgat
NFAT5	*NFAT*	Nuclear factor of activated T-cells	-128	-110	-	1.000	0.904	atgGGAAatcccacattt
NFκB p65	***NFKB***	**Nuclear factor kappa B/c-rel**	**-124**	**-112**	**+**	**1.000**	**0.982**	**gtgggattTTCCc**
NFκB	***NFKB***	**Nuclear factor kappa B/c-rel**	**-122**	**-110**	**-**	**1.000**	**0.891**	**atGGGAaaatccc**
IK1	*IKRS*	Ikaros zinc finger family	-120	-108	-	1.000	0.941	tcatGGGAaaatc
RBPJK	*RBPF*	RBPJ - kappa	-121	-107	-	1.000	0.965	ctcaTGGGaaaatcc
OLF1	*NOLF*	Neuron-specific-olfactory factor	-121	-99	+	1.000	0.894	ggatttTCCCatgagtctcaaaa
PHOX2	*CART*	Cart-1 (cartilage homeoprotein 1)	-111	-91	-	1.000	0.882	ctctcTAATtttgagactcat
MSX	*HOMF*	Homeodomain transcription factors	-107	-89	-	1.000	0.989	aactctcTAATtttgagac
GSH1	*HBOX*	Homeobox transcription factors	-104	-86	-	1.000	0.881	gtcaactctcTAATtttga
MYT1L	*MYT1*	MYT1 C2HC zinc finger protein	-96	-84	+	1.000	0.945	agagAGTTgactc
HOXC13	*ABDB*	Abdominal-B type homeodomain transcription factors	-88	-72	+	1.000	0.949	gactcctaaTAAAtatg
BRN3	*BRNF*	Brn POU domain factors	-88	-70	-	0.759	0.844	ctcatatTTATtaggagtc
PHOX2	*CART*	Cart-1 (cartilage homeoprotein 1)	-87	-67	+	1.000	0.881	actccTAATaaatatgagact

BRN5	*BRN5*	Brn-5 POU domain factors	-60	-38	+	0.751	0.844	gtctgtagctcATTCtgctctgg
PAX6	*PAX6*	PAX-4/PAX-6 paired domain binding sites	-47	-29	-	1.000	0.886	tggtgggctCCAGagcaga
HOMEZ	*HZIP*	Homeodomain-leucine zipper transcription factors	-30	-16	-	1.000	0.839	tgactATCGttcttg
TH1E47	*HAND*	Twist subfamily of class B bHLH transcription factors	-21	-1	+	1.000	0.958	tagtcaattCCAGaaaccgct
STAT	*STAT*	Signal transducer and activator of transcription	-20	-2	-	1.000	0.896	gcggtttctGGAAttgact

Supplemental Table 2: Single-stranded oligonucleotides used for electrophoretic mobility shift assays (EMSA).

**Oligonucleotide name**	**Oligonucleotide sequence (5`3`)**
Il-6_1_fwd	GATTTTTATCAAATGTGGGATTTTCCCATGAGT
Il-6_1_rev	ACTCATGGGAAAATCCCACATTTGATAAAAATC
Il-6_2_fwd	CTTTCGATGCTAAACGACGTCACATTGTGCAATCTT
Il-6_2_rev	AAGATTGCACAATGTGACGTCGTTTAGCATCGAAAG
Il-6_3_fwd	ATGCTCAAGTGCTGAGTCACTTTTAAAGAAAAA
Il-6_3_rev	TTTTTCTTTAAAAGTGACTCAGCACTTGAGCAT
Il-6_4_fwd	TGTGTCGTCTGTCATGCGCGCGTGCCTGCGTTTAA
IL6_4_rev	TTAAACGCAGGCACGCGCGCATGACAGACGACACA
USF consensus sequence_fwd	CACCCGGTCACGTGGCCTACACC
USF consensus sequence_rev	GGTGTAGGCCACGTGACCGGGTG
SP consensus sequence_fwd	ATTCGATCGGGGCGGGGCGAGC
SP consensus sequence_rev	GCTCGCCCCGCCCCGATCGAAT
NF-κB consensus site fwd	TCAGAGGGGACTTTCCGAGAGGCG
NF-κB consensus site rev	CGCCTCTCGGAAAGTCCCCTCTGA
AP1 consensus sequence_fwd	TGAGTCATGAGTCATGAGTCA
AP1 consensus sequence_rev	TGACTCATGACTCATGACTCA

Supplemental Table 3: Sequence of primers used to amplify the *Il-6* promoter region for generating luciferease reporter constructs, to verify the correctness of the sequence, and to perform site-directed mutagenesis**.** Underlined basepairs represent mutated region of a potential transcription factor in the *Il-6* promoter region used for site-directed mutagenesis.

**Primer name**	**Primer sequence (5`3`)**	**Application**
Il-6 promoter_fwd	CTAACTGGCCGGTACCAgacttgagcattggaggggttattc *	PCR to amplify *Il-6* promoter
Il-6 promoter_rev	GGCCAGATCTTGATATCCTCGAGagcggtttctggaattgactatcG *	PCR to amplify *Il-6* promoter
RVprimer3	CTAGCAAAATAGGCTGTCCC	Sequencing of *Il-6* promoter construct
pGL4.10_rev	CGGTAAAGGCGATGGTGC	Sequencing of *Il-6* promoter construct
Il-6_1_Mut	GATTTTTATCAAATGTGGGATGTTCCCATGAGTCT	Site-directed mutagenesis of NF-κB in Il-6_1
Il-6_2_Mut	CTTTCGATGCTAAACGATATCACATTGTGCAATC	Site-directed mutagenesis of cFos in Il-6_2
Il-6_3_Mut	CCATCAAGACATGCTCAAGTGCTGCTTCACTTTTAAAG	Site-directed mutagenesis of cFos in Il-6_3
Il-6_4_Mut (USF)	GTGTCGTCTGTCATGCGCGTATGCCTGCGTTTAAATAAC	Site-directed mutagenesis of USF in Il-6_4
Il-6_4_Mut (cFos)	GTGTGTCGTCTTACATGCGCGCGTGCCTGC	Site-directed mutagenesis of cFos in Il-6_4

* Capital letters indicate homologous overlap between the empty pGL4.10 reporter vector the *Il-6* promoter region designed to build the seamless clone using the Gibson Assembly Cloning kit according to manufacturer’s protocol (Thermofisher).
